# Impact of the Affordable Care Act on Referral to Care for People Living with HIV in Appalachia

**DOI:** 10.13023/jah.0202.05

**Published:** 2020-04-15

**Authors:** Cameron A. Wade, Timothy N. Crawford, Nicole E. Leedy, Alice C. Thornton

**Affiliations:** University of Kentucky College of Medicine; Wright State University; University of Kentucky; University of Kentucky

**Keywords:** Appalachia, HIV, Affordable Care Act, Medicaid Expansion

## Abstract

**Introduction:**

The Affordable Care Act (ACA) enacted on March 23, 2010 significantly impacted access to healthcare for people living with HIV (PLWH). Expansion of care was accomplished in three areas: eliminating exclusions for pre-existing conditions, elimination of lifetime caps on healthcare expenditures, and expansion of Medicaid eligibility.

**Purpose:**

This study evaluated the impact of state implementation of the ACA Medicaid expansion on referral to HIV care at a Ryan White federally funded clinic in Kentucky (University of Kentucky Bluegrass Care Clinic [UK BCC]).

**Methods:**

Retrospective chart review of all newly enrolled patients at the UK BCC between March 2010 and June 2017. Data included patient demographics and referral source, and were divided into two groups to compare enrollments before and after Kentucky implemented the ACA Medicaid expansion. Data were collected from 2018–2019 and analyzed in 2020.

**Results:**

Following Medicaid expansion there were statistically significant changes in the patterns of referral to federally funded HIV care. These included a significant decrease in the proportion of referrals from state and local health departments, and an increase in both proportion of referrals from outpatient clinics and transfers from different HIV care providers.

**Implications:**

These results have implications for engaging more PLWH into HIV care, particularly in states where patients have increased access to screening and assessment of risk at primary care encounters through implementation of the ACA Medicaid expansion.

## BACKGROUND

The Affordable Care Act (ACA) enacted on March 23, 2010, significantly affected the access to health care for people living with HIV (PLWH). Expansion of care was accomplished in three areas: elimination of exclusions for pre-existing conditions, elimination of lifetime caps on healthcare expenditures, and expansion of Medicaid eligibility.^1^ The Medicaid expansion provision in the ACA permits states to voluntarily provide expanded Medicaid benefits for individuals at or below 138% of the federal poverty level with federal funding for 3 years after enactment. To date, 36 states and the District of Columbia have adopted this provision.

The Commonwealth of Kentucky was one of the early adopters of expanded Medicaid, with expansion occurring in January of 2014. It was one of the few southern states to adopt Medicaid expansion. Following the Kentucky Medicaid expansion, the uninsured rate fell from 14.3% in 2013 to 5.4 in 2017.^2^ By the end of June 2017, the total number of diagnosed PLWH in Kentucky was 6836.^3^ With the changes in coverage for individuals in Kentucky, it was suspected that patterns of initiation to HIV care would change.

The University of Kentucky Bluegrass Care Clinic (UK BCC) is a federally funded Ryan White HIV/AIDS clinic that serves 63 counties and over 1600 PLWH in Central and Eastern Kentucky. The UK BCC receives Ryan White HIV/AIDS Program (RWHAP) funds (Parts B, C, D, and F). This study evaluated the impact of the implementation of ACA Medicaid expansion in Kentucky on referral to HIV care at UK BCC.

## METHODS

Data were abstracted from the CareWare database (Health Resources and Services Administration’s electronic health and social support services information system) used to store patient insurance information. This study examined all newly enrolled adult patients to the UK BCC from March 24, 2010 to June 8, 2017. Data were collected from 2018 to 2019 and analyzed in 2020. Data for each patient included age at time of enrollment, gender, race, HIV risk factor(s), and referral type. Referral type was further categorized into one of nine groups: referral by self, primary care provider or other outpatient clinic, from a hospitalization, from an OB/GYN, community organization, transfer from another HIV care provider, health department, referral from the UK BCC itself (includes patients who were screened in the clinic), and unknown.

The data for all patients were divided into two groups, those who were enrolled in the UK BCC before January 1, 2014 (before Kentucky implemented the ACA Medicaid expansion), and those who enrolled at or after January 1, 2014. The Pearson’s Chi-Squared Test was used to observe differences in pre-ACA and post-ACA data by demographics and referral source. In addition, a t-test was used to examine differences in age. SAS version 9.4 (Cary NC) was used to analyze all data, and a p≤0.05 was considered significant.

## RESULTS

There were 985 adult enrollments to the UK BCC for HIV care during the study period, with 407 (41.3%) between March 24, 2010 and December 31, 2013, and 578 (58.7%) between January 1, 2014 and June 8, 2017. Demographics of the patient population in these two time periods are summarized in Table 1. The two time periods were not significantly different based on age at time of intake, gender, race, or HIV risk factor; however, the two groups did vary significantly when comparing referral to care.

**Figure 1:**
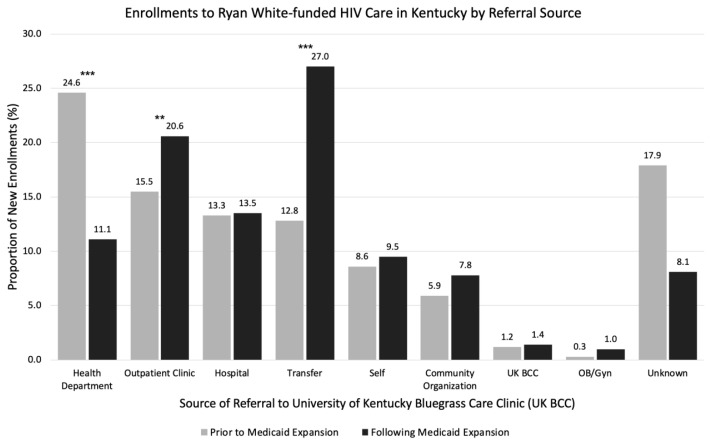
Enrollments to Ryan White–Funded HIV Care in Kentucky by Referral Source (N=985) ***p<0.0001 **p<0.05

On statistical analysis, there was an overall significant difference in referral patterns between the two time periods when considering all referral groups (p<0.0001). A visual summary of the changes in referral to care at UK BCC is made in Figure 1. Of note, there was a 13.5% decrease in the frequency of referrals from Health Departments (24.6% vs. 11.1%; p<0.0001), and a 14.2% increase in the frequency of transfers of HIV care to the UK BCC from an established HIV care provider (12.8% vs. 27.0%; p<0.0001). In addition, there was a 4.9% increase in referrals from outpatient clinics (15.5% vs. 20.6%; p=0.04). Of the 985 intake records, 120 patients had an unknown referral source. Before the ACA Medicaid expansion in Kentucky, 47% of UK BCC enrollments in this study were uninsured, compared to 5% among UK BCC enrollments following ACA Medicaid expansion.

## IMPLICATIONS

This retrospective study was an analysis of the demographics and referral source for PLWH in Central and Eastern Kentucky to a Ryan White–funded HIV clinic (UK BCC), and how those parameters differed before and after state implementation of the ACA Medicaid expansion.

Following the Kentucky Medicaid expansion there was significant change in the referral sources for new enrollments to the UK BCC. Of note there was a large decrease in the frequency of patients referred from state and local health departments, and a large increase in patient transfers from a different HIV care provider.

The decrease in referrals from Health Departments may indicate that PLWH have more access to screening and referrals via outpatient clinics through primary care providers since Medicaid expansion, particularly when considering that the uninsured proportion of new enrollments dropped from 47% to 5%. It is unclear why patients transferred to the UK BCC from existing HIV care; this would be a subject for future study and should include whether transfers were the result of changes in eligibility at their prior provider, moving from a state for coverage, or other reasons for transfer.

This study is limited by incomplete assessment of other time-varying factors that could account for changes in referral patterns, including but not limited to the implementation of education programming for outpatient providers, testing and counselling programs throughout the states, and changes to Health Department funding during the study period. Also, these data represent referral patterns to a single Ryan White clinic in a Medicaid expansion state and may not be generalizable to other similar clinics. However, the authors suspect that these results have implications for engaging more PLWH into HIV care, particularly in states that have increased access to primary care through healthcare expansion.

**SUMMARY BOX**

**What is already known about this topic?** Medicaid expansion in Kentucky substantially dropped the uninsured rate, but the impact of these policy changes has not been well characterized for people living with HIV.

**What is added by this report?** This study assessed the impact of Medicaid expansion on referral to HIV care at a federally funded clinic and found that following expansion, a smaller proportion of referrals came from Health Departments, and more from outpatient clinics or other HIV care providers.

**What are the implications for public health practice, policy, and research?** This would lead one to surmise that screening and assessment of risk has increased at primary care encounters rather than health departments, an important change for the crucial first step in initiating PLWH to care.

## Figures and Tables

**Table 1:** Study Demographics and Referral Source Among Newly Enrolled Patients 2010–2017 (N=985)

	Pre-ACA		Post-ACA		p
N	407		578		
**Age** – mean (SD)	38.1	11.6	38.0	12	0.88
**Gender**	**N**	**Percent**	**N**	**Percent**	0.38
M	334	82.1	468	81.0	
F	67	16.5	106	18.3	
Trans (MtF)	6	1.5	4	0.7	
**Race**					0.06
White (Non-Hispanic)	255	62.7	377	65.2	
Black or African American	106	26.0	144	24.9	
Hispanic	37	9.1	42	7.3	
American Indian/Alaska Native	0	0.0	3	0.5	
Pacific Islander	0	0.0	3	0.5	
Asian	1	0.3	6	1.0	
Unreported	8	2.0	3	0.5	
**HIV Risk Factor**					0.12
MSM	252	61.9	350	60.6	
IDU	13	3.2	31	5.4	
MSM and IDU	20	4.9	23	4.0	
Heterosexual	96	23.6	138	23.9	
Perinatal	6	1.5	2	0.4	
Transfusion	4	1.0	6	1.0	
Unspecified	1	0.3	0	0.0	
**Referral Type**	**N**	**Percent**	**N**	**Percent**	<0.0001
Health Department	100	24.6	64	11.1	
Outpatient Clinic	63	15.5	119	20.6	
Hospital	54	13.3	78	13.5	
Transfer	52	12.8	156	27.0	
Self	35	8.6	55	9.5	
Community Organization	24	5.9	45	7.8	
UK BCC	5	1.2	8	1.4	
OB/Gyn	1	0.3	6	1.0	
Unknown	73	17.9	47	8.1	
